# Reproductive factors and metabolic syndrome among Chinese women aged 40 years and older

**DOI:** 10.1111/1753-0407.13342

**Published:** 2022-12-16

**Authors:** Ling Bai, Xi Yang, Ziyi Sun, Zuojie Luo, Li Li, Xinghuan Liang, Jia Zhou, Liheng Meng, Yang Peng, Yingfen Qin

**Affiliations:** ^1^ Department of Cardiology The First Affiliated Hospital of Guangxi Medical University Nanning China; ^2^ Guangxi Key Laboratory Base of Precision Medicine in Cardio‐cerebrovascular Diseases Control and Prevention and Guangxi Clinical Research Center for Cardio‐cerebrovascular Diseases Guangxi Medical University Nanning China; ^3^ Department of Endocrinology The First Affiliated Hospital of Guangxi Medical University Nanning China; ^4^ Department of Occupational and Environmental Health School of Public Health, Guangxi Medical University Nanning China

**Keywords:** menopausal, metabolic syndrome, parity, reproductive factors, 绝经期, 代谢综合征, 胎次, 生殖影响因素

## Abstract

**Background:**

The aim of this study is to explore the relationship between reproductive variables and the prevalence of metabolic syndrome (MetS) and its components among Chinese women aged 40 years and older.

**Methods:**

A cross‐sectional study was conducted among 4453 women aged 40 years and older in Guangxi, China. The associations between women's reproductive factors and MetS were analyzed using a logistic regression model.

**Results:**

The prevalence of MetS was 23.9% in this population. Women with MetS were mostly older, more likely to be postmenopausal, and had higher parity. Compared to women with one prior live birth, those with three or more live births had the highest odds of having MetS (odds ratio [OR] = 1.56; 95% CI, 1.23–1.99). Similarly, compared to premenopausal women, postmenopausal participants had higher odds of having MetS (OR = 1.86; 95% CI, 1.49–2.31). No associations were observed between MetS and abortion or with age at menarche.

**Conclusions:**

Our study suggests that multiparity and menopausal status may be associated with the development of MetS. The inconsistency seen in epidemiological research to date calls for further investigation.

## Graphical abstract

**Highlights**

- Reproductive variables are associated with metabolic syndrome (MetS) and its components, but the results are conflicting.
- In this cross‐sectional study involving 4453 women over 40, we found that reproductive variables, including menarche, pregnancy, childbirth, breastfeeding, and menopause, may play a role in modulating the risk of MetS.
- Simple information on reproductive history could help identify women who might have a higher risk of metabolic disorder in their later life and prevent the subsequent related chronic diseases.

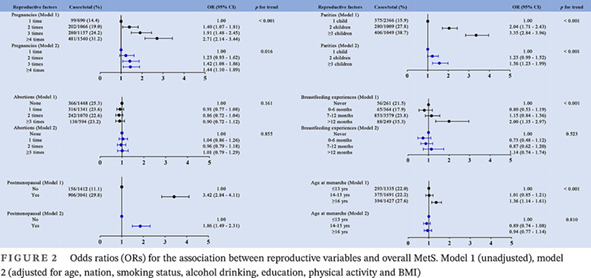

## 摘要

**目的:** 探讨中国40岁及以上妇女代谢综合征(MetS)患病率及其组成因素与生殖变量的关系。

**方法:** 对中国广西4453名40岁以上的女性进行横断面研究。使用logistic回归模型分析女性生殖影响因素与MetS之间的关系。

**结果:** 在该人群中, MetS的患病率为23.9%。患有MetS的女性大多年龄较大, 更有可能是绝经后, 且具有较多的胎次。与之前有1次活产的妇女相比, 那些≥3次活产的妇女发生MetS的几率最高(OR = 1.56;95%CI, 1.23‐1.99)。同样, 与绝经前女性相比, 绝经后参与者发生MetS的几率更高(OR = 1.86;95% ci, 1.49‐2.31)。未观察到MetS与流产或月经初潮年龄之间的关联。

**结论:** 我们的研究表明多胎和绝经状态可能与MetS的发展有关。迄今流行病学研究中发现的不同结果需要进一步调查。

## 1 INTRODUCTION

Metabolic syndrome (MetS) is a cluster of conditions occurring concomitantly, including abdominal obesity, elevated fasting blood glucose, hypertriglyceridemia, hypertension, and low high‐density lipoprotein cholesterol (HDL‐C) levels. Its incidence parallels with obesity and type 2 diabetes (T2DM), increasing the risk for atherosclerosis and cardiovascular disease (CVD).^1^, ^2^ MetS affects approximately 24.5% of the Chinese population, which is close to the global morbidity (20%–25%), and is still on the rising trend.^3^ The prevalence of MetS varies with ethnicity and gender and is also highly influenced by age.^4^, ^5^ MetS incidence is reported to be slightly higher in women (36.8%) compared to men (31.0%).^6^ Despite a disparity in terms of exposure to known risk factors that can partially explain these data, it has been hypothesized that changes in hormone levels in the reproductive stages of women might account for this unbalance.^7^

Pregnancy, in particular, is a period when the levels of estrogen and progesterone increase rapidly along with significant changes in the physiological functions of women's bodies, some of which may lead to a higher risk of adverse metabolic health outcomes in their later life.^8^ Recent studies have shown that female pregnancy‐related factors are often accompanied by the future changes of MetS and its corresponding components, but the conclusions are still conflicting. For example, the link between multiparity and a higher risk of MetS has attracted much attention,^9^, ^10^ while a recent cohort study suggested only a weak or null association.^11^ Associations between MetS and breastfeeding were positive,^12^, ^13^ but a few studies reported no association at all.^14^ The relationship between miscarriage and MetS may be due to the increased anxiety and psychological stress during miscarriage, but evidence to determine the association of miscarriage is scarce.^15^, ^16^, ^17^ While earlier age at menarche would indicate an increased chance of metabolic disorder,^18^, ^19^, ^20^ a cross‐sectional study has reported no association between the age at menarche and MetS.^21^ These inconsistent findings may have been due to the residual confounding of these reproductive factors, which were not completely ruled out by those studies.

Menopause, a phase when ovarian function declines and estrogen levels drop,^22^, ^23^ is thought to be a major factor in the rise in the occurrence of MetS in women over the age of 39.^24^, ^25^ Postmenopausal status was identified as an independent risk factor for MetS and its components.^26^ Survey data showed that compared with women aged 20–39, menopausal women aged 40–59 years were three times more likely to meet the MetS criteria, and postmenopausal women over 60 years were six times more likely to have MetS.^27^

To our knowledge, only a few studies have focused on every aspect of the reproductive factors or all individual components of MetS, even though previous studies revealed that the reproductive stage affects women's long‐term metabolic health.^28^, ^29^, ^30^ Moreover, the physiological period is a continuous process during which hormone metabolism levels in the body are constantly changing.^31^, ^32^ Given that glycolipid metabolism is often coexisting and interacting, ignoring the potential connection between each MetS component may have an impact on the overall outcomes.^33^ Furthermore, data are required to confirm the relationships between these variables and MetS and its components because the effects of reproductive factors on maternal metabolic health are still uncertain. The relationship between reproductive factors (such as parity, abortion, and duration of reproductive years) and MetS has been previously reported among Chinese women, although most of these reports focused on women aged 40 years and older.^34^, ^35^, ^36^ It could be that MetS is much more common in Chinese women (27.0%) than it is nationwide or globally, and compared with people aged 15–39, those aged 40–59 years were two times more likely to meet the MetS criteria (13.9% vs. 26.4%).^3^ China has gradually become an aging society, and every year increase in age is associated with a 3.7% increase in the prevalence of MetS, implying that the burden of metabolic diseases in the Chinese elderly population is getting heavier.^37^ And with the implementation of China's three‐child policy, the number of pregnant women is increasing year by year, so more attention needs to be paid to women's reproductive health.^38^ In this study, we utilized the cross‐sectional baseline data from a large population‐based cohort study to explore the associations between reproductive factors and MetS and all of its individual components among Chinese women aged 40 years and older in Guangxi, China.

## 2 MATERIALS AND METHODS

### 2.1 Study participants

**FIGURE 1:**
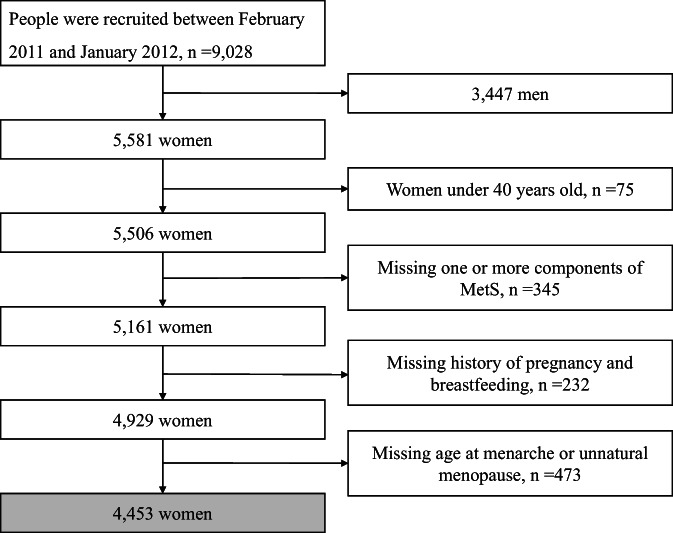
Flowchart of the study population

The cross‐sectional study subjects were recruited from Guangxi Province, China, from a nationwide sizeable prospective cohort study in China, namely the Risk Evaluation of Cancers in Chinese Diabetic Individuals: A Longitudinal Study (REACTION), which was conducted among 259 657 adults aged 40 years or older in 25 communities across mainland China during 2011–2012.^39^ The methodology of the REACTION study has been described previously. A total of 9028 participants were recruited from April 2011 to January 2012 in eight communities in Nanning, Guangxi, China. Participants meeting the following criteria were excluded: (1) men (*n* = 3447), (2) women under 40 years old (*n* = 75), (3) women without data of one or more components of MetS (*n* = 345), (4) women who did not provide a history of pregnancy or breastfeeding (*n* = 232), and (5) those women who lacked information on age at menarche or unnatural menopause (*n* = 473). A total of 4453 participants were included in the final analysis. The REACTION study was approved by the Committee on Human Research at Rui Jin Hospital, Shanghai Jiao Tong University School of Medicine and was conducted in accordance with the institutional guidelines. All study participants provided written informed consent (Figure 1).

### 2.2 Measurements and questionnaire

Participants were scheduled for a personal interview and a clinic visit after the recruitment, which took place at the First Affiliated Hospital of Guangxi Medical University. Body height, weight, waist circumference (WC), hip circumference (HC), systolic blood pressure (SBP), and diastolic blood pressure (DBP) were measured by experienced nurses according to a standard protocol. Body mass index (BMI) was calculated as body weight in kilograms divided by height in meters squared (kg/m^2^). WC was measured at an umbilical level in the standing position. An automated electronic device (OMRON Model HEM‐752 FUZZY, Omron Company, Dalian, China) was used to measure SBP and DBP in the sitting position three times consecutively at 1‐min intervals each.

During the face‐to‐face interviews, trained personnel used a standard questionnaire to collect information on sociodemographic, lifestyle factors, medical history, and family history. The levels of education were divided into high school education or above if 9 years of elementary or secondary education were completed, and less than high school if less than 9 years of elementary or secondary education were completed. Smoking and drinking status were self‐reported. Participants who had regularly smoked one cigarette per day or seven per week during the past 6 months were considered current smokers. The type and frequency of alcohol consumption were recorded, and current drinkers were defined as those who had regularly consumed alcohol once per week during the past 6 months. We categorized current and former smokers as having smoked, and the same applies to drinking history. The questionnaire estimated physical activity during work and leisure time by collecting intensity, duration, and frequency of physical activity.

### 2.3 Biochemical evaluation

Blood samples were collected after an overnight fast for at least 10 h. All participants underwent an oral glucose tolerance test, and plasma glucose was obtained at 0 and 2 h during the trial. Plasma glucose concentrations were evaluated at local hospitals using the glucose oxidase or hexokinase method within 2 h after collecting blood samples under a stringent quality control mechanism. Fasting insulin, total cholesterol (TC), low‐density lipoprotein cholesterol (LDL‐C), HDL‐C, and triglycerides (TG) were measured with an autoanalyzer (Abbott Laboratories, Illinois). The level of glycosylated hemoglobin (HbA1c) was determined by high‐performance liquid chromatography (BIO‐RAD, Hercules, California) using the VARIANT II Hemoglobin Testing System certified by the National Glycohemoglobin Standardization Program. After obtaining fasting plasma glucose (FPG) and fasting serum insulin by the methods mentioned above, the fasting insulin resistance index (FIRI) was calculated.

### 2.4 Classification and definition

Reproductive factors assessed in this study included gravidity, parity, abortion, history of breastfeeding, menopausal status, and age at menarche. The number of abortions was defined by the total number of self‐reported spontaneous and induced abortions. Information on the mean duration of breastfeeding per child was calculated as the sum of the durations divided by parities. Menopausal status was defined by the following questions: “Are you still menstruating? If yes, when was the last time you had your period?” Women who answered “no” were categorized as menopausal. Age at menarche was determined by asking, “How old were you when you first got your period?”

The National Cholesterol Education Program Adult Treatment Panel III (NCEP‐ATP III) was used to define MetS in this study. This definition requires the individual to have at least three of the following risk factors^40^: (1) abdominal circumference >102 cm for men and >88 cm for women, (2) TG ≥ 1.70 mmol/L, (3) HDL‐C < 1.04 mmol/L for men and <1.30 mmol/L for women, (4) SBP ≥ 130 mm Hg or DBP ≥ 85 mm Hg or current use of antihypertensive medication, and (5) FPG ≥ 6.1 mmol/L or previously diagnosed T2DM.

We added a sensitivity analysis using the Chinese Diabetes Society (CDS) criteria. This definition requires the individual to have at least three of the following risk factors^41^: (1) BMI ≥ 25.0 kg/m^2^, (2) TG ≥ 1.70 mmol/L, (3) HDL‐C < 0.90 mmol/L for men and <1.00 mmol/L for women, (4) SBP ≥ 140 mm Hg or DBP ≥ 90 mm Hg or current use of antihypertensive medication, and (5) FPG ≥ 6.1 mmol/L or 2‐h postprandial blood glucose (2hPG) ≥ 7.8 mmol/L or previous diagnosis of T2DM. We also performed a sensitivity analysis using the International Diabetes Federation (IDF) criteria. This definition requires an increased WC (for men ≥94 cm and for women ≥80 cm) plus at least two of the following^42^: (1) FPG ≥ 5.6 mmol/L or previous diagnosis of T2DM, (2) TG ≥ 1.70 mmol/L or current use of antihyperlipidemic medication, (3) HDL‐C < 1.04 mmol/L for men and <1.30 mmol/L for women, and (4) SBP ≥ 130 mm Hg or DBP ≥ 85 mm Hg or current use of antihypertensive medication.

### 2.5 Statistical analysis

All statistical analyses were performed using SAS software version 9.4 for Windows (SAS Institute Inc., Cary, North Carolina). In the present study, gravidity was divided into four groups: 1, 2, 3, and >3 times. Parity was categorized as: none, 1, 2, and ≥3 prior live births. Breastfeeding duration was referred to as the average period each child was breastfed: never, 1–6, 7–12, and >12 months. Abortions were categorized as: never, 1, 2, and ≥3 times. The age at menarche was evaluated in years: ≤13, 14–15, and ≥16 years old. Menopausal status was categorized as “yes” and “no.”

All continuous variables are presented as means and standard deviations (± SD) or medians and interquartile ranges, and all categorical variables are expressed as numbers and proportions. A comparison of means and proportions was performed with the independent *t* test and chi‐squared test, respectively. The prevalence of MetS and its five components in different groups of reproductive variables was also assessed. Based on the findings detailed in the literature, the variables described below were considered as potential confounders and were included in our analysis: age (40–60, ≥60 years), ethnic group (Han or the Minority), smoking (yes/no), alcohol drinking (yes/no), education level (below high school and high school or above), and physical activity (never or mild and moderate or above). Detailed information on some of these variables has been described above. The missing values were categorized into an additional category in the analysis. The associations between reproductive factors and MetS and all of its individual components were analyzed using logistic regression models. We implemented two logistic regression models to assess the relationship between reproductive factors and MetS. In model 1, no covariates were adjusted. In model 2, age, ethnic group, smoking status, alcohol drinking, education, physical activity, and BMI were further adjusted. We used odds ratios (OR) and 95% confidence intervals (CI) to estimate the strength of this association.

Following the first diagnostic criteria proposed by the World Health Organization (WHO) in 1998, the NCEP‐ATP III and the IDF have successively proposed their own diagnostic criteria.^43^ Concentrating on racial difference, the CDS recommended a definition of MetS for the Chinese population.^41^ The prevalence of MetS differs according to the definition. We performed sensitivity analyses to investigate whether the strength of the association between the reproductive variables and the odds of having MetS differs according to different criteria. MetS is an independent predictor of CVD, and the prevalence for CVD increased with increasing number of MetS components.^44^ MetS has also been reported to increase the risk of T2DM.^45^ Therefore, we had added a sensitivity analysis excluding CVD (including myocardial infarction, coronary heart disease, and hypertension) and T2DM to minimize the potential confounding effects.

The prevalence of MetS was highly influenced by age, and data showed that compared with women aged 40–59 years, women over 60 years were twice more likely to meet the MetS criteria.^27^ We conducted a subgroup analyze to divide the participants into two groups: 40–59 years old and over 60 years old. Obesity is an important part of the metabolic abnormal cluster, and overweight and obesity have also been reported to increase the risk of metabolic abnormalities.^46^ As the Working Group on Obesity in China recommended BMI cutoffs of 24.0 kg/m^2^ to define overweight and 28.0 kg/m^2^ to define obesity, we chose the cutoffs of 24.0 kg/m^2^ as the grouping basis.^47^ We conducted a subgroup analysis to divide the participants into two groups: BMI ≥ 24 kg/m^2^ and BMI < 24 kg/m^2^.

## 3 RESULTS

### 3.1 General and reproductive factors of participants

Among the 4453 women eligible for the analysis (mean [SD] age, 56.7 [10.7] years, 1698 [38.1%] aged ≥60 years), 1062 women reported MetS. Table 1 shows the demographic characteristics and biochemical indicators of the participants in this study. MetS women were characterized by older age (59.2% vs. 40.8%), more significant proportions of Han (73.9% vs. 22.7%), less likely to get high school or above degrees (66.8% vs. 32.6%), less likely to do physical exercise with medium or high intensity (23.5% vs. 74.8%), and less likely to have smoked (0.9% vs. 93.7%) or drank (14.8% vs. 80.6%) in the past. They also had lower HDL‐C levels and higher BMI, FIRI, WC, BP, HC, FPG, 2hPG, TC, TG, and LDL‐C levels.

**TABLE 1:** Characteristics of the participants by MetS

	Total	MetS	No MetS	*p* values
*n*	4453	1062	3391	
Age, *n* (%)				<0.001
40–60	2755 (61.9)	433 (40.8)	2322 (68.5)	
≥ 60	1698 (38.1)	629 (59.2)	1069 (31.5)	
Ethnic group, *n* (%)				<0.001
Han	3010 (67.6)	785 (73.9)	2225 (65.6)	
Minority	1327 (29.8)	241 (22.7)	1086 (32.0)	
Missing	116 (2.6)	36 (3.4)	80 (2.4)	
Smoking, *n* (%)				0.909
Yes	38 (0.9)	10 (0.9)	28 (0.8)	
No	4169 (93.6)	995 (93.7)	3174 (93.6)	
Missing	246 (5.5)	57 (5.4)	189 (5.6)	
Alcohol, *n* (%)				<0.001
Yes	840 (18.9)	157 (14.8)	683 (20.1)	
No	3408 (76.5)	856 (80.6)	2552 (75.3)	
Missing	205 (4.6)	49 (4.6)	156 (4.6)	
Education, *n* (%)				<0.001
Below high school	2468 (55.4)	709 (66.8)	1759 (51.9)	
High school or above	1967 (44.2)	346 (32.6)	1621 (47.8)	
Missing	18 (0.4)	7 (0.7)	11 (0.3)	
Physical activity, *n* (%)				<0.001
Never or mild	3119 (70.0)	794 (74.8)	2325 (68.6)	
Moderate and above	1242 (27.9)	249 (23.5)	993 (29.3)	
Missing	92 (2.1)	19 (1.8)	73 (2.2)	
Age, years, mean ± SD	56.7 ± 10.6	61.8 ± 10.0	55.1 ± 10.2	<0.001
SBP, mm Hg, mean ± SD	131.9 ± 20.4	144.3 ± 17.6	128.0 ± 19.6	<0.001
DBP, mm Hg, mean ± SD	77.5 ± 11.0	82.0 ± 11.1	76.1 ± 10.6	<0.001
BMI, kg/m^2^, mean ± SD	23.9 ± 3.3	26.1 ± 3.4	23.2 ± 3.0	<0.001
WC, cm, mean ± SD	81.3 ± 8.9	88.6 ± 8.3	79.1 ± 7.7	<0.001
HC, cm, mean ± SD	93.7 ± 6.8	97.2 ± 7.1	92.6 ± 6.3	<0.001
FPG, mmol/L, mean ± SD	5.8 ± 1.5	6.8 ± 2.2	5.5 ± 1.0	<0.001
2hPG, mmol/L, mean ± SD	7.8 ± 3.4	10.1 ± 4.4	7.0 ± 2.6	<0.001
HbA1c, %, mean ± SD	5.8 ± 1.0	6.4 ± 1.3	5.6 ± 0.8	<0.001
TC, mmol/L, mean ± SD	5.2 ± 1.4	5.5 ± 1.3	5.1 ± 1.4	<0.001
TG, mmol/L, mean ± SD	1.5 ± 1.1	2.4 ± 1.6	1.2 ± 0.7	<0.001
HDL‐C, mmol/L, mean ± SD	1.4 ± 0.4	1.2 ± 0.3	1.5 ± 0.4	<0.001
LDL‐C, mmol/L, mean ± SD	3.1 ± 1.0	3.2 ± 1.0	3.0 ± 1.0	<0.001
FIRI, mU/L, mean ± SD	9.3 ± 7.7	12.9 ± 11.7	8.1 ± 5.5	<0.001

Abbreviations: 2hPG, 2‐hour postprandial blood glucose; BMI, body mass index; DBP, diastolic blood pressure; FIRI, fasting insulin resistance index; FPG, fasting plasma glucose; HbA1c, glycosylated hemoglobin; HC, hip circumference; HDL‐C, high‐density lipoprotein cholesterol; LDL‐C, low‐density lipoprotein cholesterol; MetS, metabolic syndrome; SBP, systolic blood pressure; TC, total cholesterol; TG, triglycerides; WC, waist circumference.

### 3.2 Prevalence of MetS and its individual components according to reproductive factors

**FIGURE 2:**
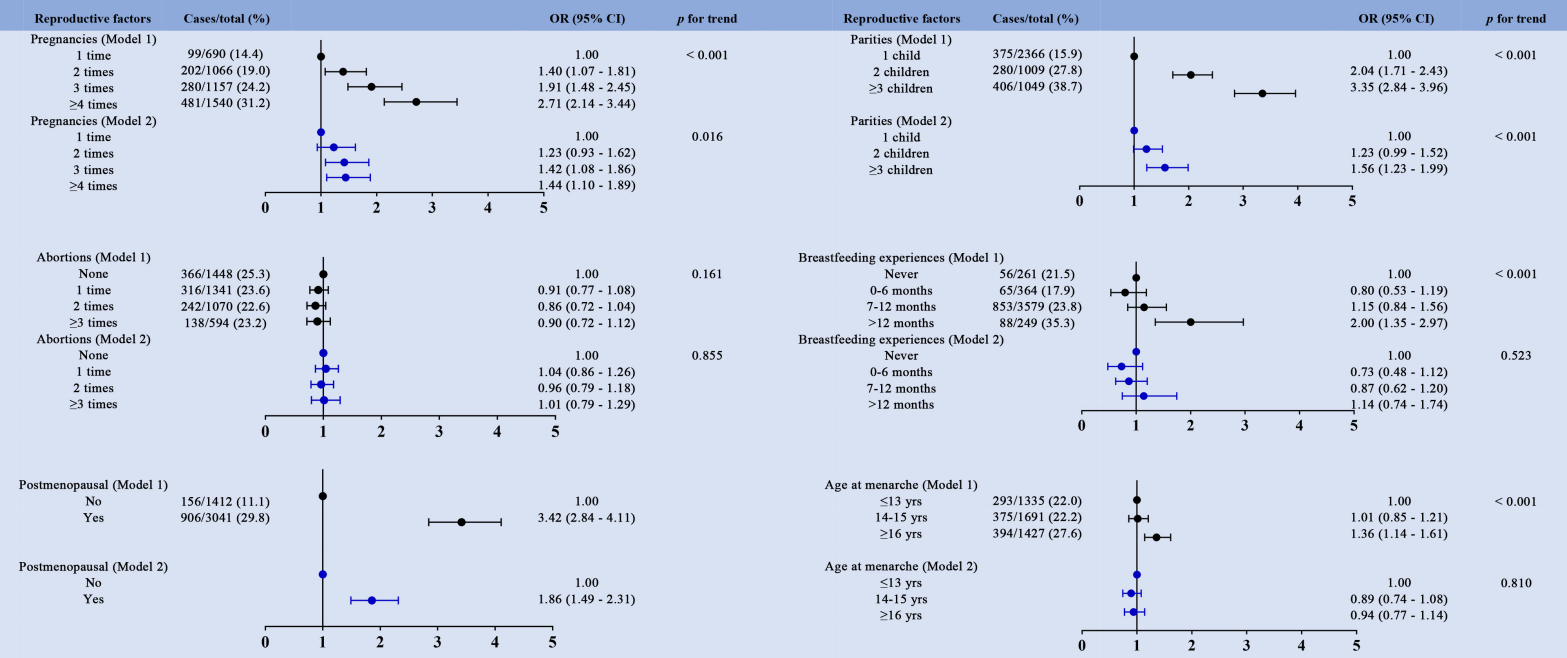
Odds ratios (ORs) for the association between reproductive variables and overall MetS. Model 1 (unadjusted), model 2 (adjusted for age, nation, smoking status, alcohol drinking, education, physical activity and BMI)

The relationship between reproductive variables and the prevalence of MetS as well as its individual components is presented in Figure 2 and Figure [Link], [Link]. Among the included participants, elevated BP was the most prevalent MetS component with a prevalence estimation of 55.3%, while abdominal obesity was the least prevalent component with prevalence estimates of 20.7%. The prevalence of MetS varied significantly and showed an increasing trend with increase of pregnancies, more prior live births, longer breastfeeding duration, and earlier age at menarche. The number of MetS components also showed the same trend as the prevalence of MetS. Among the participants, postmenopausal women were more likely to have abdominal obesity compared to premenopausal women. Moreover, women who gave birth to three or more children (37.5%), those who reported having breastfed longer (total > 12 months) (38.6%), and those who had a later menarche age (26.1%) were also likely to suffer from abdominal obesity. Compared with the group of two live births (30.0%), the prevalence of elevated TG increased in the group of three or more parities (31.4%). In addition, the prevalence of elevated TG was highest in the group that had breastfed more than 12 months compared to the group that had breastfed less time or those who had never breastfed. Women with low HDL‐C were less likely to be menopausal (35.5% vs. 45.8%). However, we did not find any trend for the occurrence of low HDL‐C between the groups with pregnancies or breastfeeding duration. Participants with age at menarche ≥16 years more often reported an elevated BP than women with an earlier age at menarche. In the group of participants with elevated BP levels, there were fewer premenopausal women and a higher proportion of those with three or more live births. The prevalence of fasting glucose impairment showed the same trend with elevated BP in all groups.

### 3.3 Association between reproductive factors and MetS and its components

We utilized multivariate logistic regression analysis to evaluate the association between reproductive factors and MetS and its components. Compared to the women with one pregnancy, those who had three pregnancies had approximately 42% higher odds of having MetS (OR = 1.42; 95% CI, 1.08–1.86; Figure 2), and those who had more than four pregnancies had 70% higher odds of having an elevated abdominal circumference (OR = 1.70; 95% CI, 1.25–2.32; Figure S1). We also found that women who had more than four pregnancies had higher odds of having an elevated BP (OR = 1.27; 95% CI, 1.04–1.56; Figure S4) and fasting blood glucose (OR 1.42; 95% CI, 1.08–1.86; Figure S5). The group with more than three parities was found to have higher odds of having MetS (OR = 1.56; 95% CI, 1.23–1.99; Figure 2) and elevated abdominal circumference (OR = 1.93; 95% CI, 1.48–2.53; Figure S1) than the one‐parity group in both models as well as the highest odds of having an elevated BP (OR = 1.31; 95% CI, 1.06–1.63; Figure S4) and fasting blood glucose (OR = 1.63; 95% CI, 1.28–2.08; Figure S5) after adjusting for the potential confounders mentioned above. Subjects in the group that had breastfed 12 months or more had approximately 65% higher odds of having elevated abdominal circumference (OR = 1.65; 95% CI, 1.01–2.69; Figure S1) compared with those in the group who had never breastfed. There were no links found between miscarriage and MetS or its separate components. When compared with premenopausal women, postmenopausal women had higher odds of having MetS (OR = 1.86; 95% CI, 1.49–2.31; Figure 2) and elevated abdominal circumference (OR = 1.91; 95% CI, 1.48–2.46; Figure S1), TG (OR = 1.78; 95% CI, 1.47–2.16; Figure S2), BP (OR = 2.18; 95% CI, 1.86–2.55; Figure S4), fasting blood glucose (OR = 1.59; 95% CI, 1.28–1.98; Figure S5), as well as reduced HDL‐C (OR = 0.58; 95% CI, 0.50–0.68; Figure S3). Age at menarche ≥16 years was associated with significantly greater odds of having MetS in unadjusted analyses (OR = 1.36; 95% CI, 1.14–1.61; Figure 2). We also found a similar pattern in abdominal obesity (OR = 1.63; 95% CI, 1.36–1.96; Figure S1), elevated BP (OR = 1.65; 95% CI, 1.42–1.92; Figure S4), and abnormal fasting glucose (OR = 1.38; 95% CI, 1.15–1.66; Figure S5), whereas age at menarche ≥16 years was associated with marginally lower odds of having reduced HDL‐C (OR = 0.81; 95% CI, 0.70–0.95; Figure S3) in nonadjusted analyses. The associations were attenuated in fully adjusted models.

### 3.4 Association between reproductive factors and MetS in sensitivity analysis and subgroup analyses

In the overall population, OR of Mets (defined according to IDF criteria) was enhanced by reproductive factors (Figure S8). Results from this sensitivity analysis were similar to the results of the main analysis, mainly in that multiparity and menopausal status were associated with an increased prevalence of MetS, whereas subjects in the group of those who had breastfed less than 6 months had approximately 43% lower odds of having MetS (OR = 0.63; 95% CI, 0.43–0.92) compared with the those who had never breastfed. There was no sign of a relationship between miscarriage and MetS. The same observations were noted when the CDS criteria of MetS were applied (Figure S7). Multiparity and menopausal status often meant higher odds of having MetS. In the population excluding CVD and T2DM, the results also tended in the same direction as in the primary analysis (Figure S6).

In the subgroup of women aged 40–59, multiparity and menopausal status were linked to significantly greater odds of having MetS (Figure S9‐1). Compared to women with one pregnancy, those who had three or more than four pregnancies had higher odds of having MetS, which were 1.59 (95% CI, 1.15–2.20) and 1.48 (95% CI, 1.06–2.08), respectively, whereas in the subgroup of women over 60 years, the group with more than three parities was found to have higher odds of having MetS (OR = 1.51; 95% CI, 1.08–2.12) (Figure S9‐2). In women with BMI ≥ 24 kg/m^2^, that is, the overweight or obese subgroup, the results also tended in the same direction as in the primary analysis (Figure S10‐2), whereas in the subgroup of women with BMI < 24 kg/m^2^, we found no links between multiparity and MetS (Figure S10‐1). When compared with those who had never breastfed, subjects in the group of those who had breastfed less than 6 months had lower odds of having MetS (OR = 0.49; 95% CI, 0.24–0.96).

## 4 DISCUSSION

To our knowledge, this epidemiological study is the most extensive investigation of multiple reproductive factors concerning the development of MetS among Chinese women. Reproductive events throughout a woman's life, including menarche, pregnancy, childbirth, breastfeeding, and menopause, may potentially promote later‐life MetS through pathways involving hormonal and metabolic alterations.^36^, ^48^ In this cross‐sectional study, we confirmed that female reproductive variables were significantly related to the prevalence of MetS and its individual components. Our results imply that multiparity was associated with an increased prevalence of selected MetS components, particularly abdominal obesity, elevated BP, and elevated fasting glucose, after controlling for sociodemographic and behavioral characteristics. A similar trend was also exhibited in the multipregnancy and menopause groups, but no significant relationships were found between the other three reproductive variables and MetS.

Previous research on the association between multiparity and specific MetS components has yielded mixed results. A cross‐sectional study by Wu et al. showed that parity correlated with 52% higher odds of having MetS in women with four or more live births.^10^ Multiparity is assumed to cause metabolic problems by initiating insulin resistance and inducing the formation of intra‐abdominal adipose tissue.^49^ Similarly, we observed a 56% increase in the prevalence of MetS among those with three or more live birth. Two other Chinese investigations have supported the positive link between multiparity and MetS.^20^, ^34^ In contrast, Shi et al. suggested that high parity had no positive or negative relationship with MetS.^50^ Thus, more research is needed to substantiate this link.

A meta‐analysis found breastfeeding to be a powerful protector against MetS, especially in terms of lipid levels and WC, with a greater protective impact than on other components.^51^ Two Korean studies have also shown that postpartum breastfeeding prevents women from developing MetS later in life.^13^, ^52^ However, we found no evidence that linked breastfeeding to a reduced incidence of MetS. Similar to our results, a study by Moradi et al. in Iran did not find breastfeeding to be protective against the development of MetS.^53^ Most notably, the data from our study confirmed that the duration of breastfeeding was significantly related to abdominal obesity. This result is consistent with prior research studies, which concluded that BMI and WC increased with prolonged breastfeeding.^54^

A prior study by Xu et al. showed that abortion was linked to a 25% increase in the chance of having MetS, and the correlation was number‐dependent.^35^ The hypothesis is that disrupting the endocrine processes that coexist with the fetus might have a substantial and long‐term impact on endocrinology and metabolism.^55^ Two other previous studies showed that women with recurrent abortions have greater levels of oxidative stress.^56^, ^57^ A central underlying mechanism for MetS has been identified as an increase in the generation of oxidizing species.^58^ Our data, however, did not support this connection.

The variations of estrogen levels owing to changes in ovarian function, which most studies have attributed as the primary reasons for metabolic abnormalities in women, might be linked to the age at menarche and menopausal status.^19^, ^59^ Evidence suggests that women who had their menarche at a younger age have a higher risk of obesity, and childhood obesity has been associated with the development of MetS in adulthood.^18^, ^60^ On the other hand, our data revealed no association between age at menarche and MetS and only a weak nonsignificant correlation between later menarche age and low HDL‐C. That is likely due to the fact that the average age of the participants in our research was rather young (mean age = 56.7 years old). In terms of menopausal state, our findings are in line with some other studies reporting that the prevalence of MetS and its components was much more significant in postmenopausal women.^61^, ^62^ Menopause means not only the loss of reproductive potential but also an increased susceptibility to metabolic diseases, which are often accompanied by an increase in central adiposity, particularly visceral fat.^63^ Meanwhile, higher risks of significant CVD and all‐cause mortality also have been observed among postmenopausal women.

Overall, our findings suggest that reproductive variables may play a vital role in the development of MetS. This study builds on previous research by investigating reproductive characteristics and all individual MetS components in Chinese women aged 40 and above. The large cohort recruited from the REACTION study, which has a thorough training procedure and quality assurance processes, is one of our study's strengths. This is the first study investigating the interaction between reproductive variables and MetS and its individual components in the Chinese Guangxi community.

Our study has several limitations that should be addressed since they may impact the conclusions drawn from the findings. First, this cross‐sectional study did not evaluate the chronological order of occurrence of the relevant factors, thus, potentially affecting the outcome. Second, as our study only included those 4453 women over 40 from Guangxi, the findings may not apply to the broader domestic community as well as to other ethnicities worldwide. Third, given we did not know the participants' exact ages each time they gave birth, we could not judge the impact of their reproductive years on this study. We also did not collect related medical history such as gestational hypertension and gestational diabetes mellitus, which may also affect metabolic health later in life. Fourth, we lacked information on emotions, lifestyle, and stress in relation to MetS after each birth, which could lead to residual confounding and weaken the observed association. Because the subjects we recruited were only from a single hospital, population selection bias may have occurred. Therefore, extrapolating our findings to other populations should be done with caution.

## 5 CONCLUSIONS

Among reproductive factors investigated in the current study, multiparity and menopausal status were found to be related to MetS and its components after adjusting some confounding factors. Given the expected growth of our country's middle‐aged and elderly population, appropriate exercise and attention to reproductive health can be recommended to reduce the risk of MetS later in life. Many controversies exist about the relationship between MetS and other reproductive factors such as abortions; hence, comprehensive research using longitudinal and multidimensional approaches is needed to evaluate the independent association of each factor with metabolic health. Simple information on reproductive history could help identify women with a higher risk of metabolic disorders later in life. Knowledge of reproductive health can contribute to the recognition of early signs and preventing MetS risk factors and subsequent chronic diseases.

## AUTHOR CONTRIBUTIONS

Ling Bai, Yang Peng and Ziyi Sun designed the study, conducted the evaluation, and designed the tables and figures. Yingfen Qin, Xi Yang, Zuojie Luo, Li Li, Xinghuan Liang, Jia Zhou, and Liheng Meng collected the data. Ling Bai wrote the first draft with contributions from Yang Peng, Xi Yang, and Yingfen Qin. All authors reviewed and revised the subsequent drafts of the manuscript.

## ACKNOWLEDGEMENTS

We thank the Department of Endocrinology of the First Affiliated Hospital of Guangxi Medical University for providing the data for this study. This work was supported by the National Key Research & Development Plan for Precision Medicine Key Program (2016YFC0901200, 2016YFC0901205), the National Health Commission of China Public Welfare Research Project (201502007), the “Excellent Medical Talent” Research and Innovation Ability Training Project of the First Affiliated Hospital of Guangxi Medical University (2017025), and the Innovation Project of Clinical Research Climbing Plan of the First Affiliated Hospital of Guangxi Medical University (YYZS2020012).

## CONFLICT OF INTEREST

The authors have no conflict of interest relevant to the article.

## Supporting information


**Figure S1.** Odds ratios (ORs) for the association between reproductive variables and abdominal obesity. Model 1 (unadjusted), model 2 (adjusted for age, nation, smoking status, alcohol drinking, education, physical activity and BMI).

**Figure S2.** Odds ratios (ORs) for the association between reproductive variables and elevated TG. Model 1 (unadjusted), model 2 (adjusted for age, nation, smoking status, alcohol drinking, education, physical activity and BMI).

**Figure S3.** Odds ratios (ORs) for the association between reproductive variables and low HDL‐C. Model 1 (unadjusted), model 2 (adjusted for age, nation, smoking status, alcohol drinking, education, physical activity and BMI).

**Figure S4.** Odds ratios (ORs) for the association between reproductive variables and elevated blood pressure. Model 1 (unadjusted), model 2 (adjusted for age, nation, smoking status, alcohol drinking, education, physical activity and BMI).

**Figure S5.** Odds ratios (ORs) for the association between reproductive variables and impaired fasting glucose. Model 1 (unadjusted), model 2 (adjusted for age, nation, smoking status, alcohol drinking, education, physical activity and BMI).

**Figure S6.** Odds ratios (ORs) for the association between reproductive variables and overall MetS after exclusion for CVD and T2DM participants. Model 1 (unadjusted), model 2 (adjusted for age, nation, smoking status, alcohol drinking, education, physical activity and BMI).

**Figure S7.** Odds ratios (ORs) for the association between reproductive variables and overall MetS using the CDS criteria. Model 1 (unadjusted), model 2 (adjusted for age, nation, smoking status, alcohol drinking, education, physical activity and BMI).

**Figure S8.** Odds ratios (ORs) for the association between reproductive variables and overall MetS using the IDF criteria. Model 1 (unadjusted), model 2 (adjusted for age, nation, smoking status, alcohol drinking, education, physical activity and BMI).

**Figure S9.** Odds ratios (ORs) for the association between reproductive variables and overall MetS in the subgroup analysis by age. Model 1 (unadjusted), model 2 (adjusted for age, nation, smoking status, alcohol drinking, education, physical activity and BMI). **Figure S9‐1.** Aged 40 to 59 years **Figure S9‐2.** Over 60 years old

**Figure S10.** Odds ratios (ORs) for the association between reproductive variables and overall MetS in the subgroup analysis by BMI. Model 1 (unadjusted), model 2 (adjusted for age, nation, smoking status, alcohol drinking, education, physical activity and BMI). **Figure S10‐1.** BMI < 24 kg/m^2^ **Figure S10‐2.** BMI ≥24 kg/m^2^
