# Supplementary figures and images for: Reproductive factors and metabolic syndrome among Chinese women aged 40 years and older
Source: J Diabetes. 2022 Dec 16;15(1):36–46. doi: 10.1111/1753-0407.13342 (PMC9870746; doi:10.1111/1753-0407.13342)

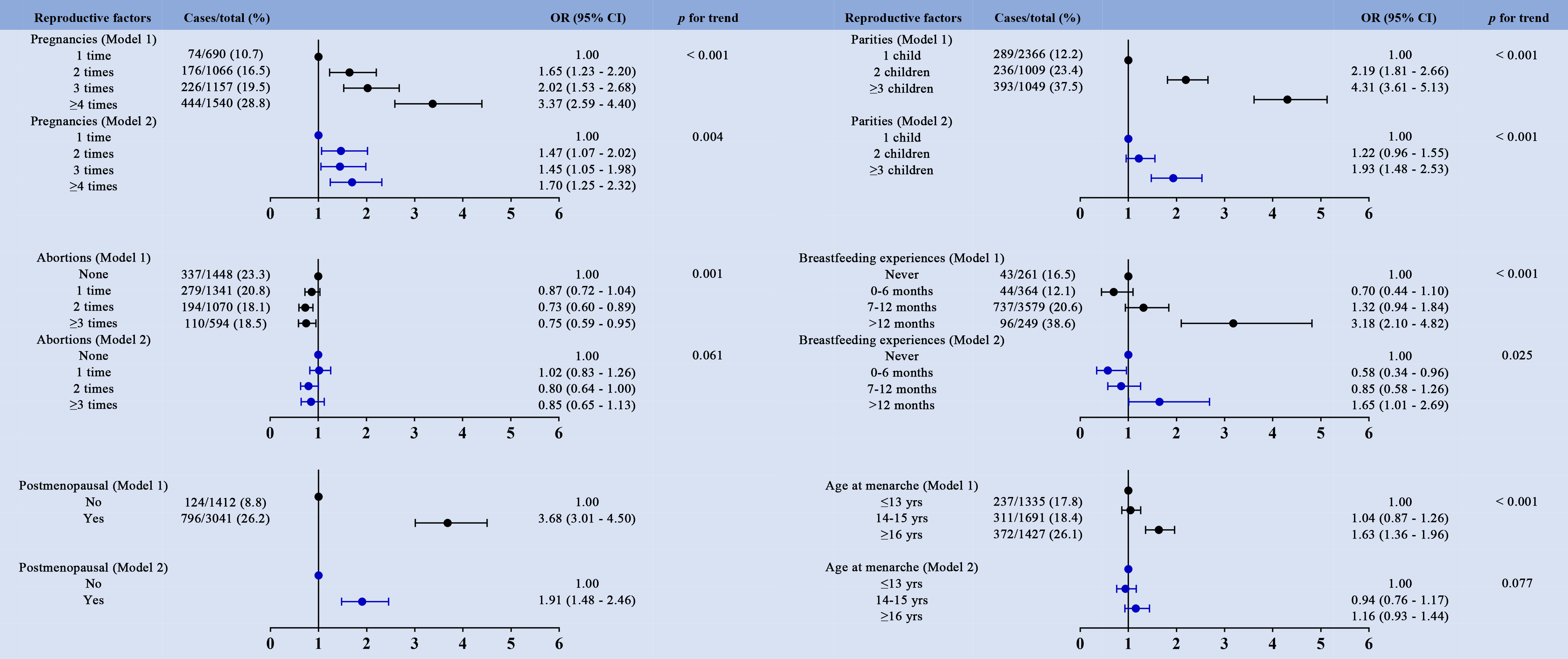

Supplement: Supplementary file 1 — Figure S1. Odds ratios (ORs) for the association between reproductive variables and abdominal obesity. Model 1 (unadjusted), model 2 (adjusted for age, nation, smoking status, alcohol drinking, education, physical activity and BMI). [file JDB-15-36-s002.png]

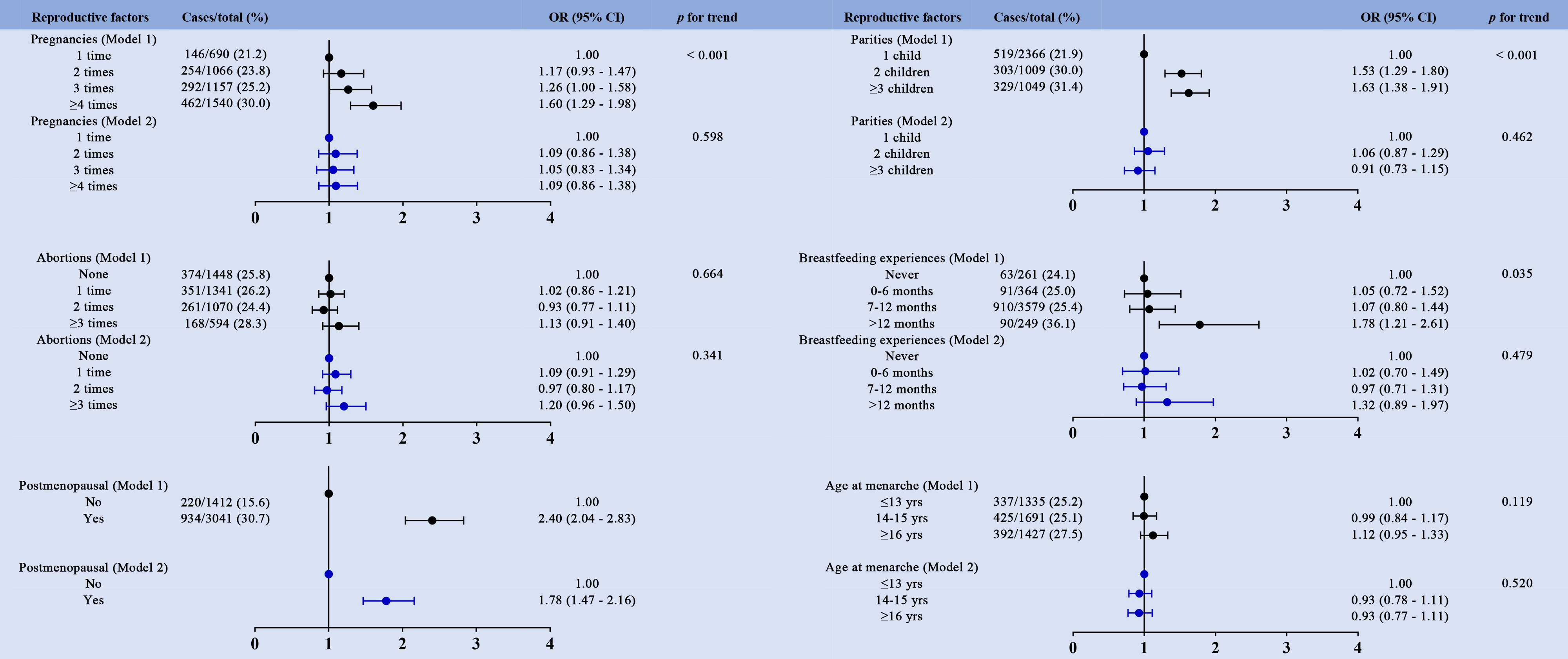

Supplement: Supplementary file 2 — Figure S2. Odds ratios (ORs) for the association between reproductive variables and elevated TG. Model 1 (unadjusted), model 2 (adjusted for age, nation, smoking status, alcohol drinking, education, physical activity and BMI). [file JDB-15-36-s007.png]

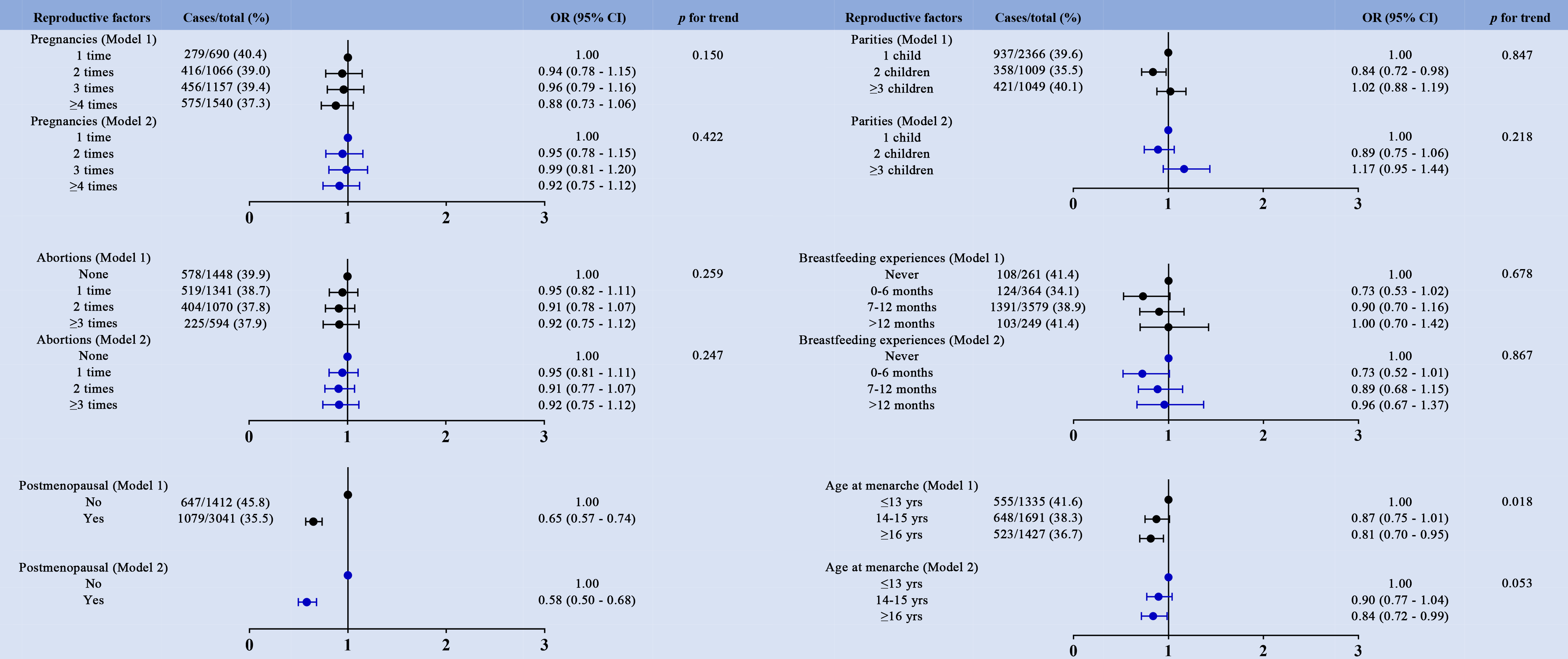

Supplement: Supplementary file 3 — Figure S3. Odds ratios (ORs) for the association between reproductive variables and low HDL‐C. Model 1 (unadjusted), model 2 (adjusted for age, nation, smoking status, alcohol drinking, education, physical activity and BMI). [file JDB-15-36-s006.png]

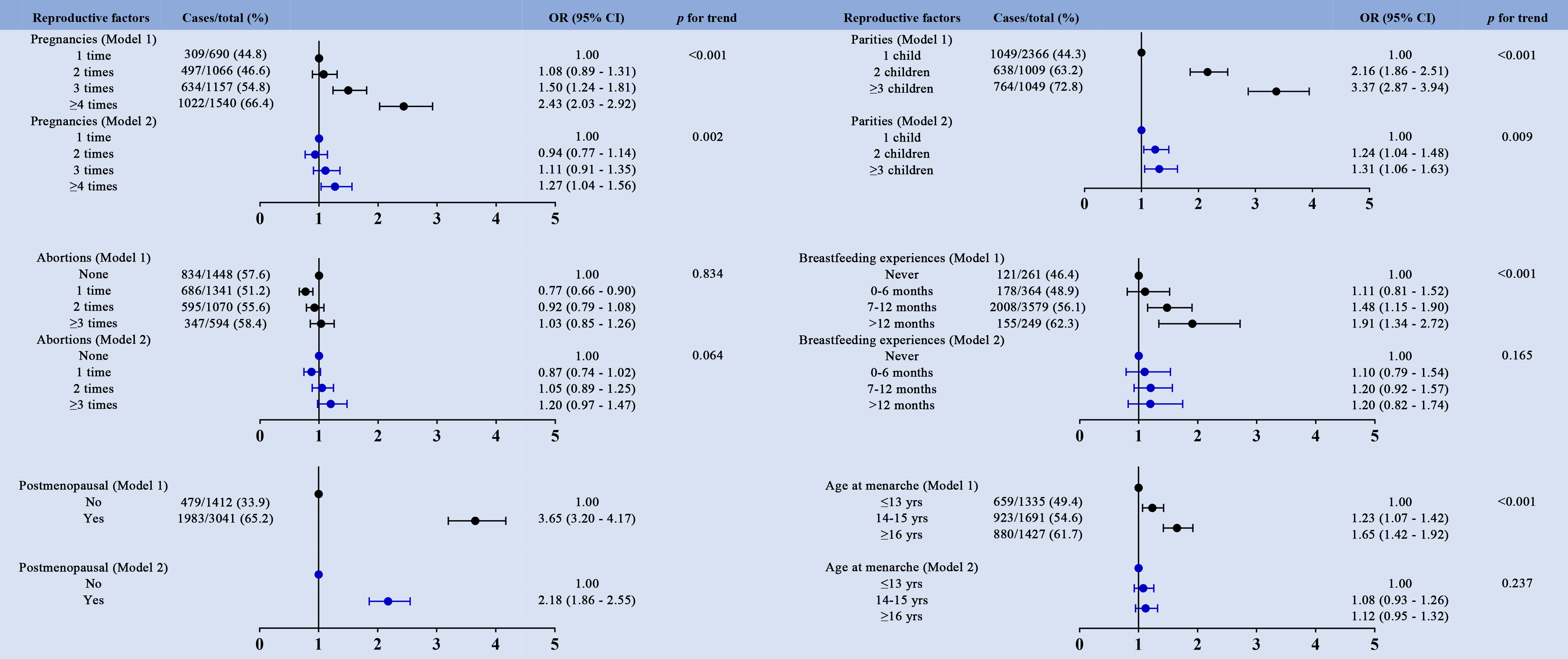

Supplement: Supplementary file 4 — Figure S4. Odds ratios (ORs) for the association between reproductive variables and elevated blood pressure. Model 1 (unadjusted), model 2 (adjusted for age, nation, smoking status, alcohol drinking, education, physical activity and BMI). [file JDB-15-36-s004.png]

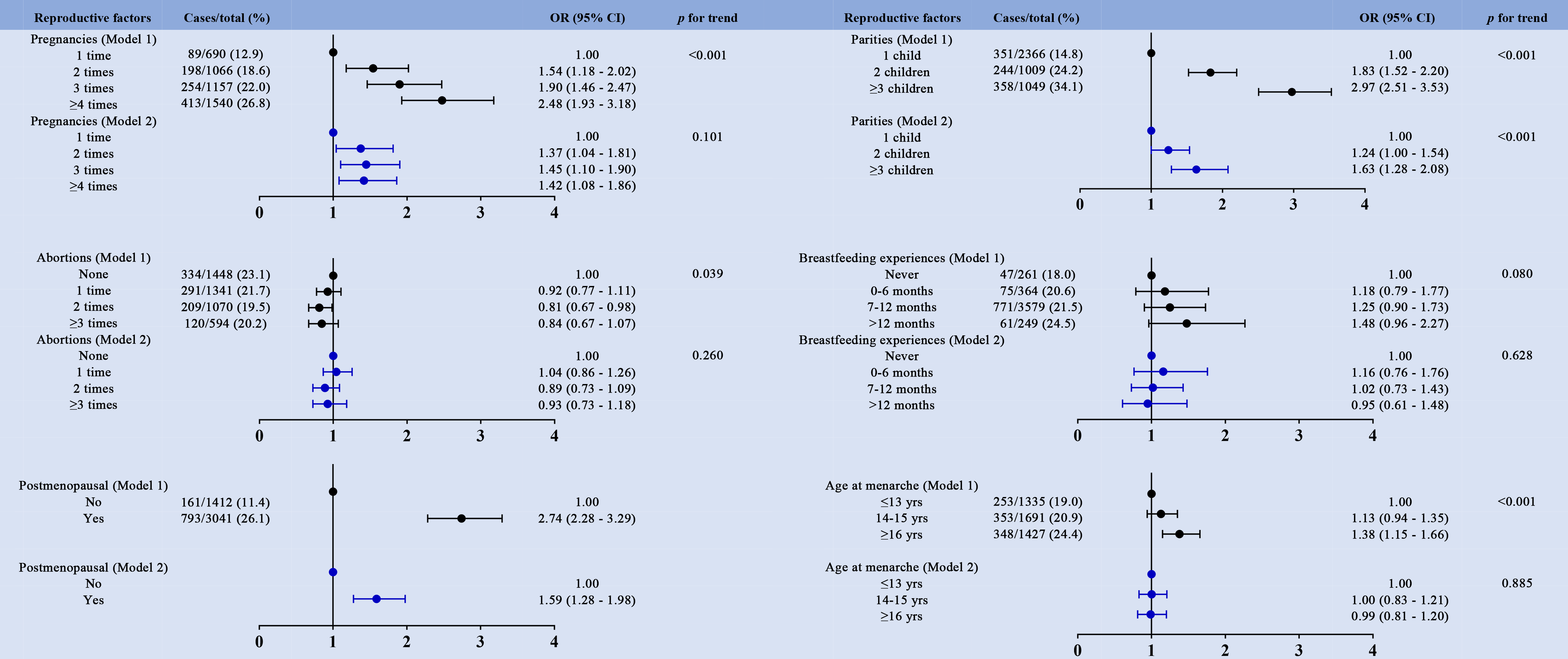

Supplement: Supplementary file 5 — Figure S5. Odds ratios (ORs) for the association between reproductive variables and impaired fasting glucose. Model 1 (unadjusted), model 2 (adjusted for age, nation, smoking status, alcohol drinking, education, physical activity and BMI). [file JDB-15-36-s008.png]

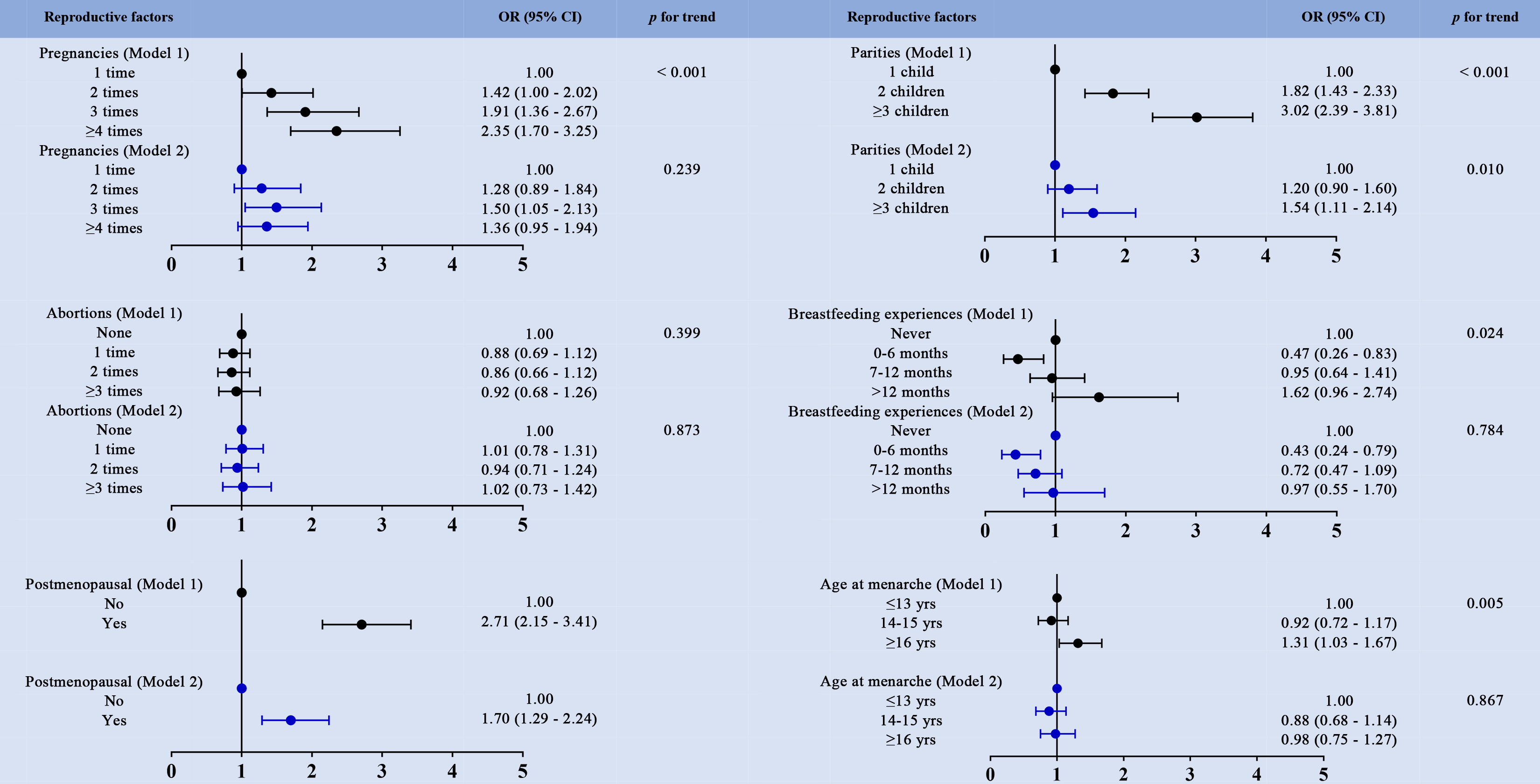

Supplement: Supplementary file 6 — Figure S6. Odds ratios (ORs) for the association between reproductive variables and overall MetS after exclusion for CVD and T2DM participants. Model 1 (unadjusted), model 2 (adjusted for age, nation, smoking status, alcohol drinking, education, physical activity and BMI). [file JDB-15-36-s003.png]

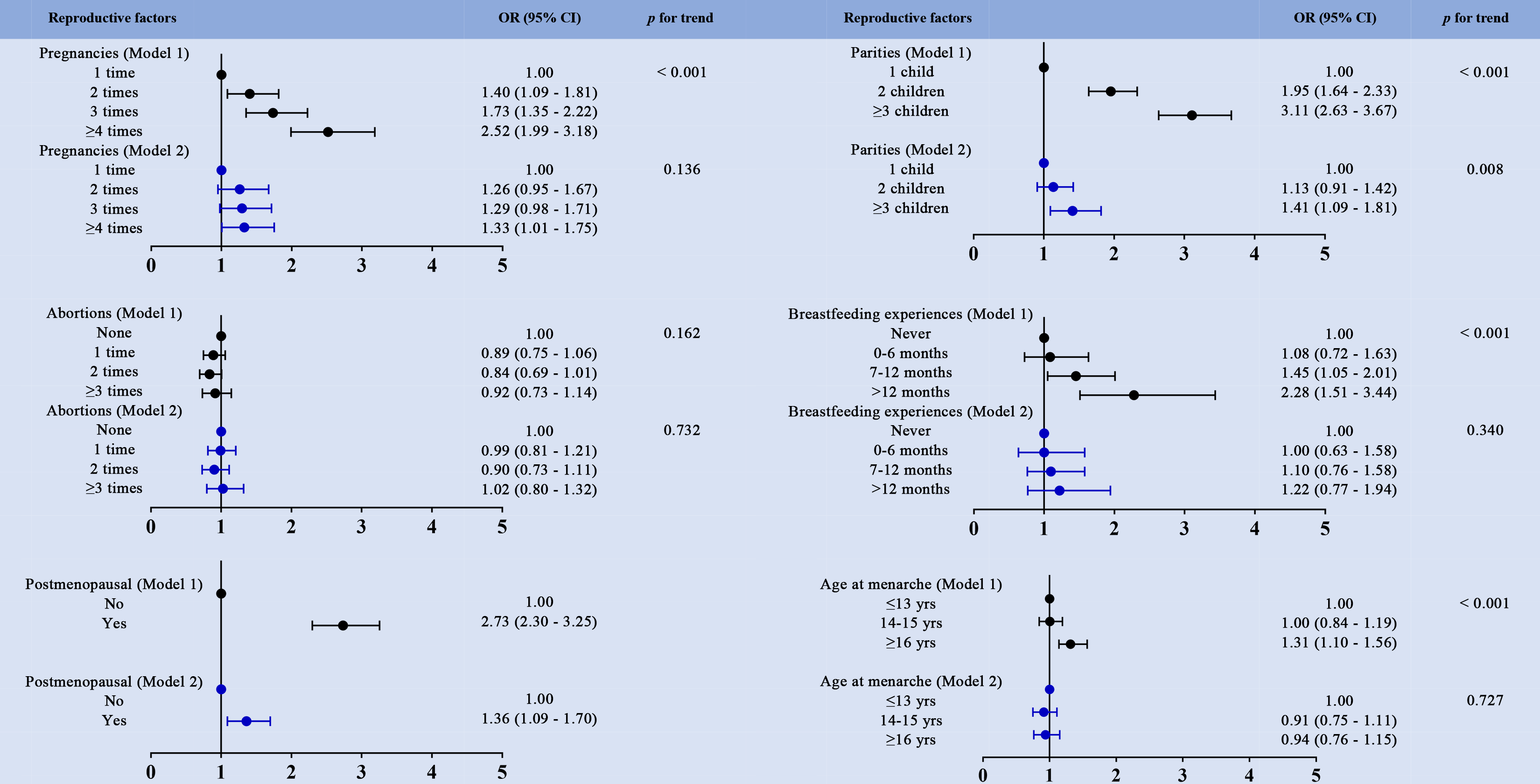

Supplement: Supplementary file 7 — Figure S7. Odds ratios (ORs) for the association between reproductive variables and overall MetS using the CDS criteria. Model 1 (unadjusted), model 2 (adjusted for age, nation, smoking status, alcohol drinking, education, physical activity and BMI). [file JDB-15-36-s001.png]

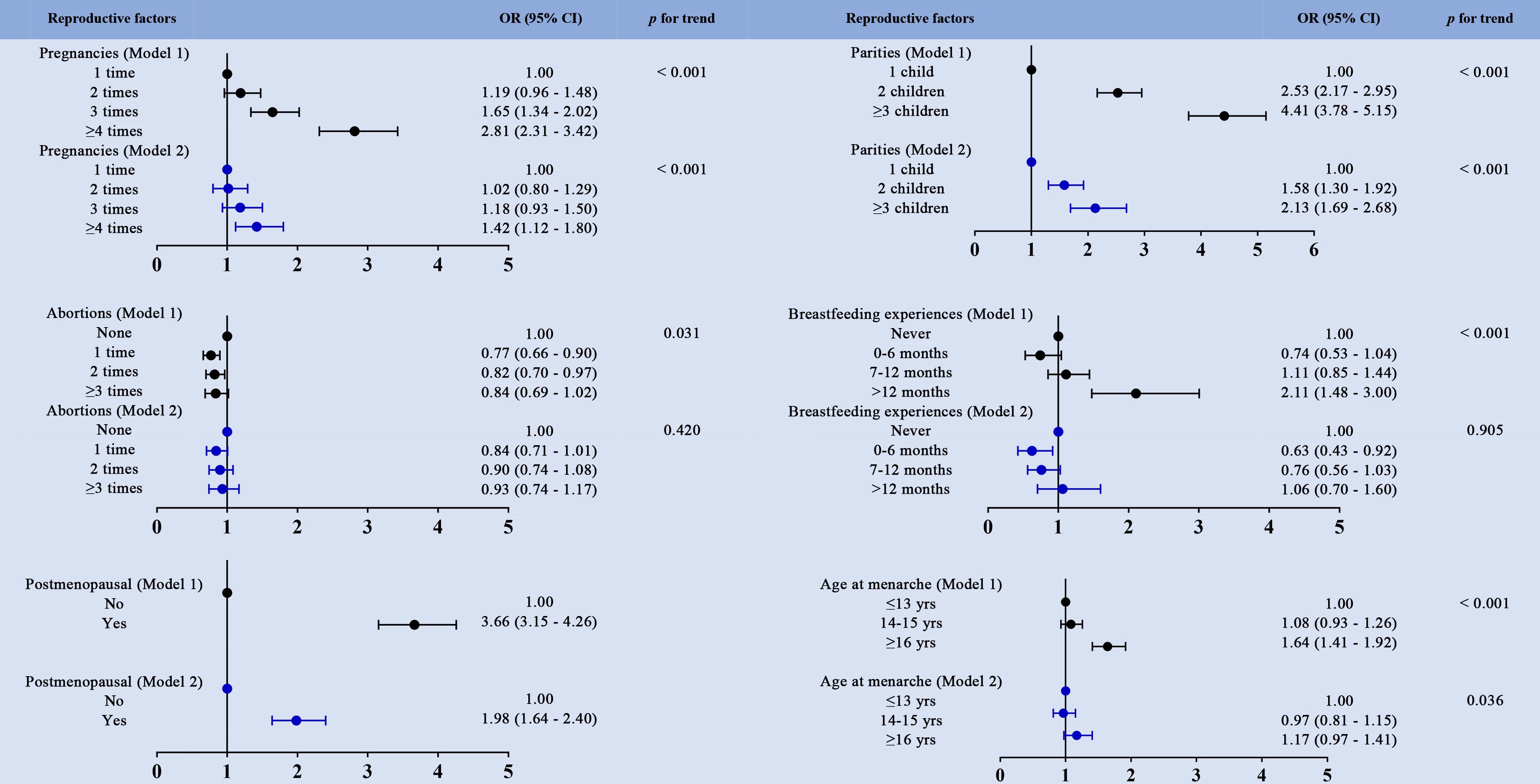

Supplement: Supplementary file 8 — Figure S8. Odds ratios (ORs) for the association between reproductive variables and overall MetS using the IDF criteria. Model 1 (unadjusted), model 2 (adjusted for age, nation, smoking status, alcohol drinking, education, physical activity and BMI). [file JDB-15-36-s005.png]

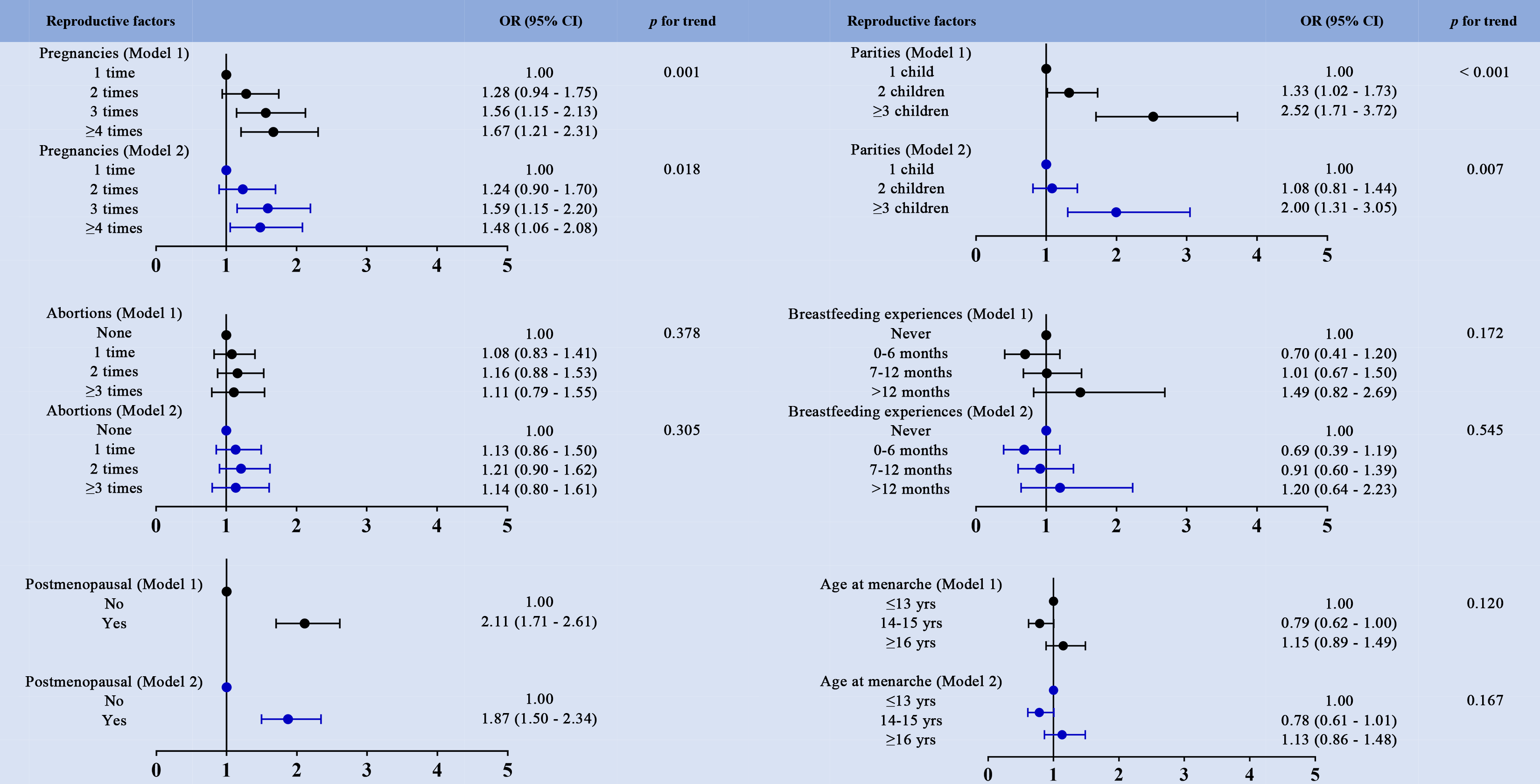

Supplement: Supplementary file 9 — Figure S9. Odds ratios (ORs) for the association between reproductive variables and overall MetS in the subgroup analysis by age. Model 1 (unadjusted), model 2 (adjusted for age, nation, smoking status, alcohol drinking, education, physical activity and BMI). Figure S9‐1. Aged 40 to 59 years Figure S9‐2. Over 60 years old [file JDB-15-36-s009.zip › JDB_13342_图11-1.png]

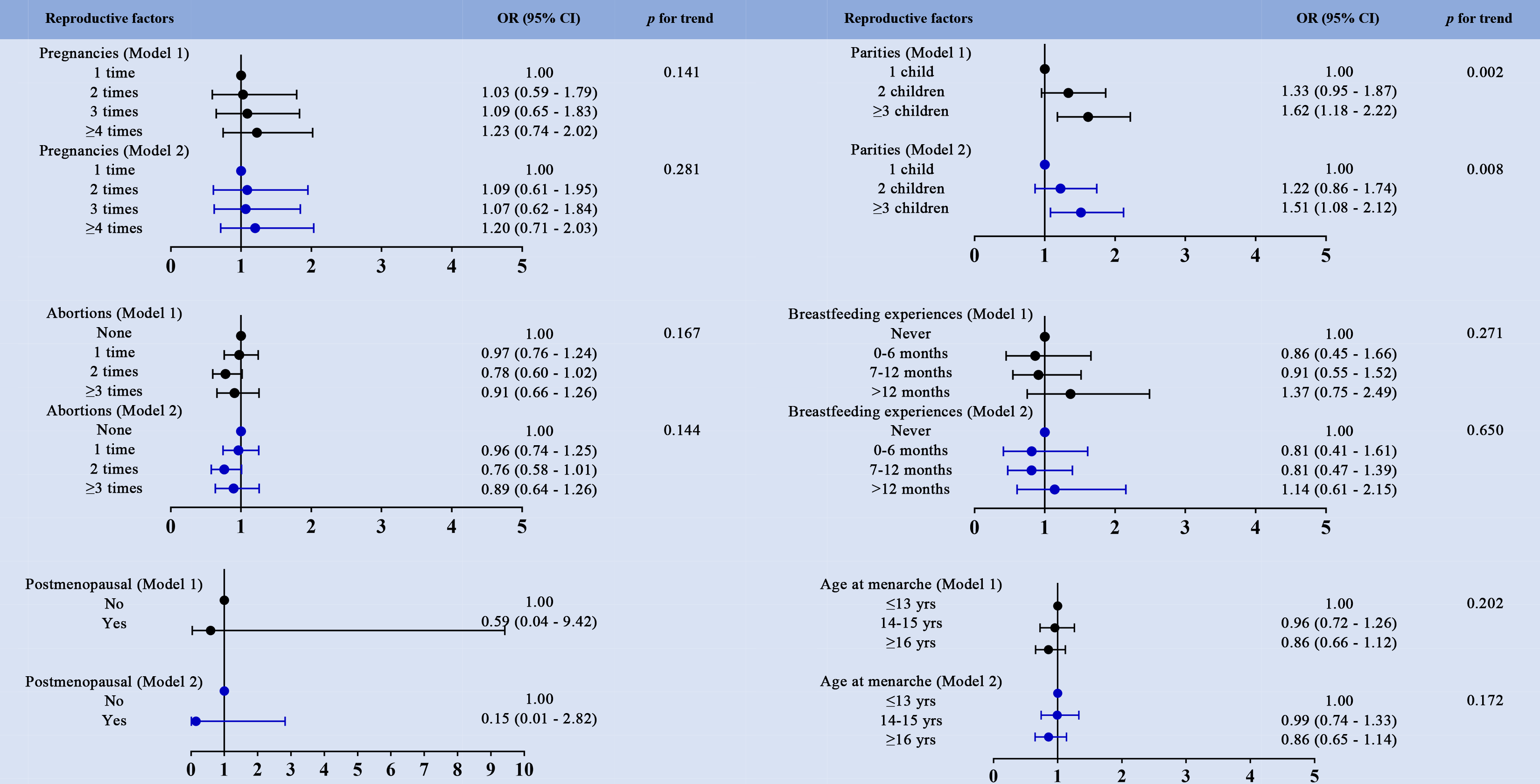

Supplement: Supplementary file 9 — Figure S9. Odds ratios (ORs) for the association between reproductive variables and overall MetS in the subgroup analysis by age. Model 1 (unadjusted), model 2 (adjusted for age, nation, smoking status, alcohol drinking, education, physical activity and BMI). Figure S9‐1. Aged 40 to 59 years Figure S9‐2. Over 60 years old [file JDB-15-36-s009.zip › JDB_13342_图11-2.png]

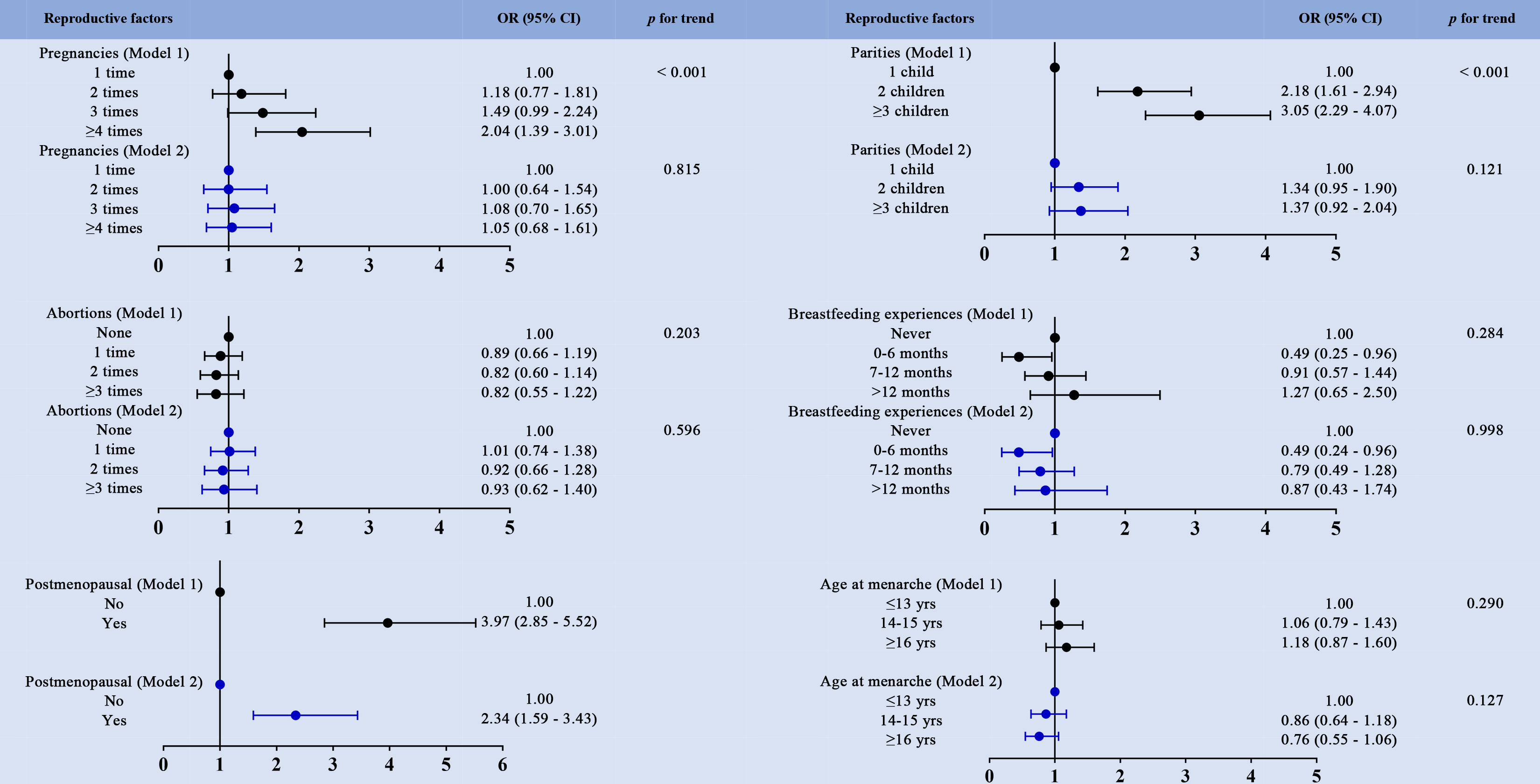

Supplement: Supplementary file 10 — Figure S10. Odds ratios (ORs) for the association between reproductive variables and overall MetS in the subgroup analysis by BMI. Model 1 (unadjusted), model 2 (adjusted for age, nation, smoking status, alcohol drinking, education, physical activity and BMI). Figure S10‐1. BMI < 24 kg/m2 Figure S10‐2. BMI ≥24 kg/m2 [file JDB-15-36-s010.zip › JDB_13342_图12-1.png]

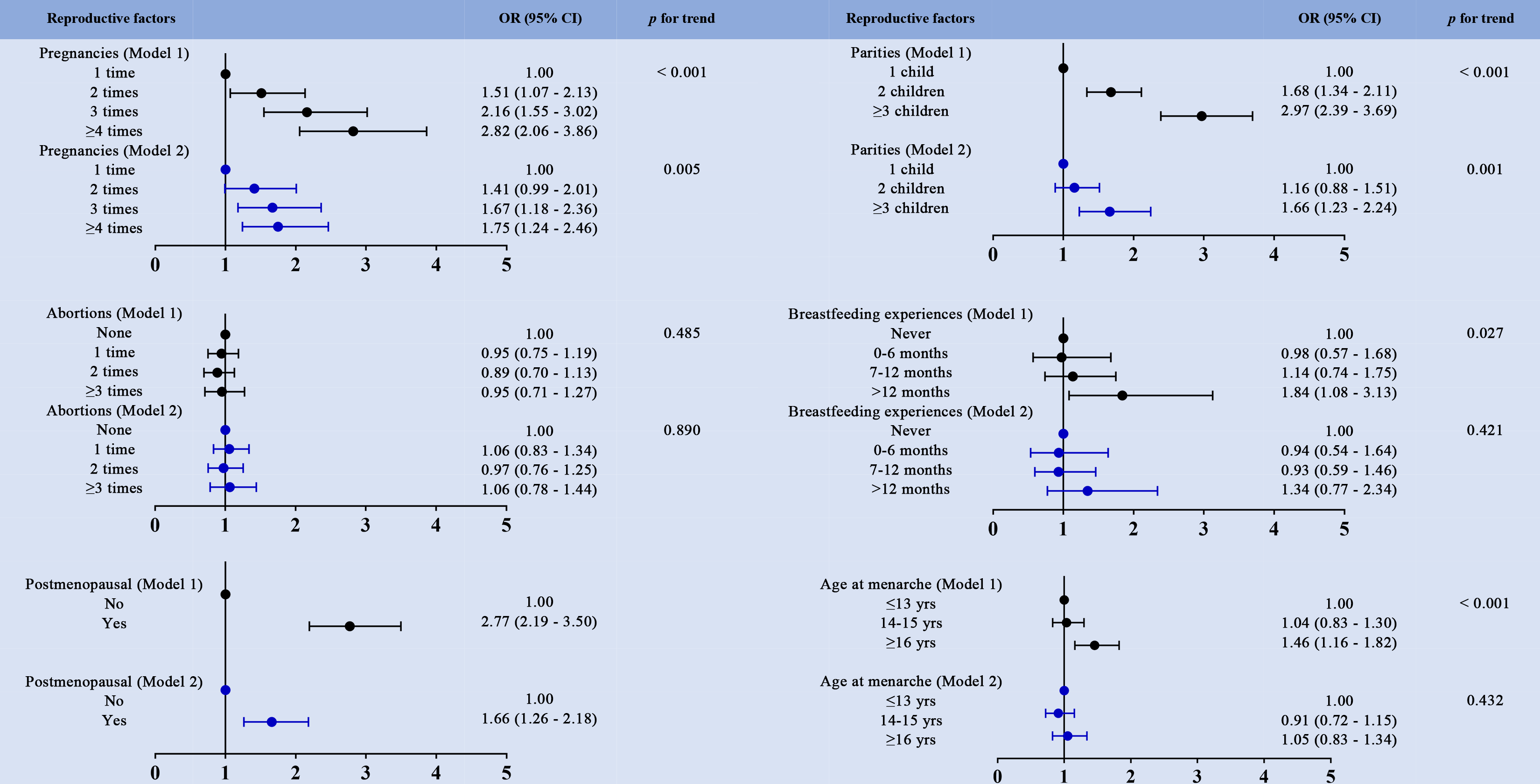

Supplement: Supplementary file 10 — Figure S10. Odds ratios (ORs) for the association between reproductive variables and overall MetS in the subgroup analysis by BMI. Model 1 (unadjusted), model 2 (adjusted for age, nation, smoking status, alcohol drinking, education, physical activity and BMI). Figure S10‐1. BMI < 24 kg/m2 Figure S10‐2. BMI ≥24 kg/m2 [file JDB-15-36-s010.zip › JDB_13342_图12-2.png]
